# Supplementary material for: Nitrogen and phosphorus losses via surface runoff from tea plantations in the mountainous areas of Southwest China
Source: PLoS One. 2023 Jun 23;18(6):e0285112. doi: 10.1371/journal.pone.0285112 (PMC10289461; doi:10.1371/journal.pone.0285112)
Supplement: S1 Table — (DOCX) [file pone.0285112.s001.docx]

**Table S1.** **Characteristics of annual rainfall events and erosion runoff events during the observation period.**

| Years | Rainfall | | Erosion runoff | |
| --- | --- | --- | --- | --- |
|  | Events | Amount (mm) | Events | Amount (mm) |
| 2018-2019 | 185 | 1262 | 45 | 1001 |
| 2019-2020 | 113 | 996 | 32 | 761 |
| 2018-2020 | 298 | 2258 | 77 | 1762 |
