# Supplementary material for: Nitrogen and phosphorus losses via surface runoff from tea plantations in the mountainous areas of Southwest China
Source: PLoS One. 2023 Jun 23;18(6):e0285112. doi: 10.1371/journal.pone.0285112 (PMC10289461; doi:10.1371/journal.pone.0285112)
Supplement: S2 Table — (DOCX) [file pone.0285112.s002.docx]

**Table S2. The difference of runoff events and erosion runoff events under different rainfall intensities in the tea plantations from September 2018 to August 2020.**

| Rainfall intensities | Rainfall | | Erosion runoff | |
| --- | --- | --- | --- | --- |
|  | Events | Amount (mm) | Events | Amount (mm) |
| Light rain | 237 | 618 | 16 | 123 |
| Moderate rain | 39 | 699 | 39 | 699 |
| Heavy rain | 18 | 647 | 18 | 647 |
| Rainstorm | 4 | 294 | 4 | 294 |
