# Supplementary material for: Nitrogen and phosphorus losses via surface runoff from tea plantations in the mountainous areas of Southwest China
Source: PLoS One. 2023 Jun 23;18(6):e0285112. doi: 10.1371/journal.pone.0285112 (PMC10289461; doi:10.1371/journal.pone.0285112)
Supplement: S3 Table — (DOCX) [file pone.0285112.s003.docx]

**Table S3. Characteristics of runoff events, runoff amounts and runoff coefficient** **during the observation period.**

| Years | Runoff events | Runoff amounts (mm) | Runoff coefficient (%) |
| --- | --- | --- | --- |
| 2018–2019 | 45 ± 0 a | 41 ± 3 a | 3.29 ± 0.25 a |
| 2019–2020 | 32 ± 0 b | 31 ± 2 b | 3.07 ± 0.22 a |
| 2018-2020 | 77 ± 0 | 72 ± 4 | 3.19 ± 0.16 |
