# Supplementary material for: Nitrogen and phosphorus losses via surface runoff from tea plantations in the mountainous areas of Southwest China
Source: PLoS One. 2023 Jun 23;18(6):e0285112. doi: 10.1371/journal.pone.0285112 (PMC10289461; doi:10.1371/journal.pone.0285112)
Supplement: S4 Table — (DOCX) [file pone.0285112.s004.docx]

**Table S4.** **The difference of** **runoff events, runoff amounts and runoff coefficient under different rainfall intensities in the tea plantations from September 2018 to August 2020.**

| Rainfall intensities | Runoff events | Runoff amounts (mm) | Runoff coefficient (%) |
| --- | --- | --- | --- |
| Light rain | 16 ± 0 c | 5 ± 1 d | 0.87 ± 0.10 d |
| Moderate rain | 39 ± 0 a | 31 ± 4 a | 4.46 ± 0.24 a |
| Heavy rain | 18 ± 0 b | 26 ± 4 b | 3.94 ± 0.20 b |
| Rainstorm | 4 ± 0 d | 10 ± 3 c | 3.42 ± 0.05 c |
