# Supplementary material for: Nitrogen and phosphorus losses via surface runoff from tea plantations in the mountainous areas of Southwest China
Source: PLoS One. 2023 Jun 23;18(6):e0285112. doi: 10.1371/journal.pone.0285112 (PMC10289461; doi:10.1371/journal.pone.0285112)
Supplement: Attachment — Submitted filename: Xing.docx [file pone.0285112.s005.docx]

Xing-cheng et al. PLOS 1.

This is an interesting study on nitrogen and phosphorus losses from tea plantations. However, I found several issues related to data processing and interpretation, statistics and writing style.

My major concerns:

1) The authors did not comprehensively explain their statistical approach in the methods section. There is no information on the statistical models, data transformation, mean separation and "pathway analysis." I was lost when they brought the results from the abovementioned analysis in the results and discussion sections. They have also used statistical terms like "significant" without presenting a test or context. Many comparisons were also made without a statistical approach. I urge authors to take a second look at their manuscript, update statistical methods and tests and base the interpretations on the statistical tests.
2) Crucial information is missing in sample collection and data processing. The authors did not mention whether the presented concentration is daily or flow-weighted.
3) Figure 3/4. Adding daily loads as a panel under one of these figures would be beneficial, and it will also help to see if large events are associated with major nutrient losses.
4) Avoid repeating results in the discussion section.
5) Avoid repeating your results in the conclusion section.

Line 34: Bouwman et al. 2002

Line 35: Phosphorus accumulation

Line 37: Please replace "chiefly" with "primarily"

Line 38: "emissions" generally indicate "gaseous discharge." Here you are mainly discussing "water discharge." Please use more appropriate terminology.

Line 55-58: Briefly discuss the findings from previous studies that assessed runoff pollution from tea plantations.

Line 76: What is the spacing between the plots?

Line 98: No "statistical tests" are mentioned here. Briefly explain data curation, distribution and transformation, statistical analysis, mean separation, and p-value for statistical significance.

Line 111: Please delete "much"

Line 115: In the methods section, please explain how you differentiated "erosive rainfall events" from "non-erosive rainfall" events.

Line 123: Please explain how you define surface runoff events in the methods section. As per your figure 3, there were periods where runoff occurred for two or more consecutive dates. Were they considered single events?

Line 133: Did you use "daily flow-weighted mean concentration" or "daily concentration"? Unclear.

Line 135: Can you support this claim with a statistical test?

Line 139: Ensure the spacing between the values and units is consistent.

Figure 4: Seasonal variation or daily variation? What do error bars represent?

Table 1. Section 2.4 statistical analysis did not provide any information about the tests you performed and mean separation methods. We can evaluate your results and interpretation without this critical information.

Also, does "daily mean concentration"?

Line 145: Provide the statistical model and p-value to support this claim.

Line 147: Wrong. Your N and P concentration were similar for light rain and rainstorm based on your statistical analysis (which we have no idea as it is not provided). Also no difference in PP concentration among all rainfall events.

Line 163: You bring this "pathway analysis" from nowhere. There is nothing written about this in the materials and methods. So many terms (e.g., action coefficient) and symbols (e.g., lambda) were being forced here without any previous explanations, which could not be very clear to readers.

Figure 5: What precipitation, event, daily or cumulative? Be specific.

Line 191: Avoid mentioning results in the discussion. Also, you cant throw these numbers without a range and context. Numbers change depending on weather, topography and management.

Line 194: Delete. You are repeating the same information in the next sentence.

Line 195: You often use "significant" without a test or supporting evidence.

Table 5: You can add the "slope/topography" of the plantations to this table, which may explain some of the observed variability.

Line 199- it was reported

Line 202- pollution from tea plantations in Southwest China

Line 210- Not entirely true. Overland flow is generated when the intensity exceeds the soil's infiltration capacity. It doesn't necessarily mean that the soils are saturated at that time. Revise.

Lines 211-213 – Merge these sentences

Line 220- Cropping systems

Line 236- delete the sentence "according to…."
