# Supplementary material for: Nitrogen and phosphorus losses via surface runoff from tea plantations in the mountainous areas of Southwest China
Source: PLoS One. 2023 Jun 23;18(6):e0285112. doi: 10.1371/journal.pone.0285112 (PMC10289461; doi:10.1371/journal.pone.0285112)
Supplement: Attachment — Submitted filename: Response to Reviewers.docx [file pone.0285112.s006.docx]

The Editor-in-chief

PLOS ONE

Dear Sir,

Responses to Reviewers comments on PONE-D-22-23175

We thank the reviewers for their overall very positive and encouraging assessment and their constructive comments, which helped us to significantly improve the manuscript. The changes are updated on the current manuscript. We trust that this version is in good shape for acceptance. Below are the detailed responses for each of the suggested changes.

| **Comment** | **Response** |
| --- | --- |
| **Reviewer 1** |  |
| 1) The authors did not comprehensively explain their statistical approach in the methods section. There is no information on the statistical models, data transformation, mean separation and "pathway analysis." I was lost when they brought the results from the abovementioned analysis in the results and discussion sections. They have also used statistical terms like "significant" without presenting a test or context. Many comparisons were also made without a statistical approach. I urge authors to take a second look at their manuscript, update statistical methods and tests and base the interpretations on the statistical tests. | We thank Reviewer for critical suggestions. The purpose of this study is to determine if the runoff, and nitrogen and phosphorus loss of tea plantation are affected by rainfall, and if the runoff, and nitrogen and phosphorus loss are significant difference under rainfall intensities. Statistical methods include variance analysis and path analysis. We have added a paragraph to Materials and Methods (line 99-103) to highlight this issue. |
| 2) Crucial information is missing in sample collection and data processing. The authors did not mention whether the presented concentration is daily or flow-weighted. | The detailed information of sample collection and data processing is supplemented in the Materials and Methods. The concentration is the daily average concentration. In this study, the runoff pond rather than the flowmeter is used as the basic unit to calculate the daily loss load. |
| 3) Figure 3/4. Adding daily loads as a panel under one of these figures would be beneficial, and it will also help to see if large events are associated with major nutrient losses. | Noted and agreed. The daily loads of total nitrogen andtotal phosphorus in surface runoff from the tea plantations have been adding in manuscript (section 3.3). |
| 4) Avoid repeating results in the discussion section. | It is well noted and rephrased to improve clarity |
| 5) Avoid repeating your results in the conclusion section. | Noted and rephrased to for clarity |
| Line 34: Bouwman et al. 2002 | noted and correction effected |
| Line 35: Phosphorus accumulation | noted and correction effected |
| Line 37: Please replace "chiefly" with "primarily" | noted and correction effected |
| Line 38: "emissions" generally indicate "gaseous discharge." Here you are mainly discussing "water discharge." Please use more appropriate terminology. | Well note, it has been rephrased for clarity |
| Line 55-58: Briefly discuss the findings from previous studies that assessed runoff pollution from tea plantations. | Well note, it has been rephrased for clarity |
| Line 76: What is the spacing between the plots? | The spacing of each plot is 6 m, which is adding in Materials and Methods |
| Line 98: No "statistical tests" are mentioned here. Briefly explain data curation, distribution and transformation, statistical analysis, mean separation, and p-value for statistical significance. | Data management, statistical analysis, mean value separation and p-value of statistical significance have been described in Materials and Methods |
| Line 111: Please delete "much" | Well note, it has been deleted |
| Line 115: In the methods section, please explain how you differentiated "erosive rainfall events" from "non-erosive rainfall" events. | We have explained the terms of “erosive rainfall events” and “non-erosive rainfall events” in the Materials and Methods section (line 99-100). |
| Line 123: Please explain how you define surface runoff events in the methods section. As per your figure 3, there were periods where runoff occurred for two or more consecutive dates. Were they considered single events? | We have explained the terms of “surface runoff events” in the Materials and Methods section (line 100). As shown in Figure 3, when runoff occurs on multiple consecutive days, it is repeatedly observed. We define it as multiple runoff events, which is helpful to clearly understand the correlation between runoff and rainfall events. |
| Line 133: Did you use "daily flow-weighted mean concentration" or "daily concentration"? Unclear. | The concentration is the daily concentration. In this study, the runoff pond rather than the flowmeter is used as the basic unit to calculate the daily loss load. |
| Line 135: Can you support this claim with a statistical test? | We have corrected the inaccurate description |
| Line 139: Ensure the spacing between the values and units is consistent. | noted and corrected |
| Figure 4: Seasonal variation or daily variation? What do error bars represent? | noted and corrected. Figure 4 describes the daily changes of total nitrogen and phosphorus concentrations in surface runoff during the monitoring period. The error bar represents the standard deviation of sample estimation |
| Table 1. Section 2.4 statistical analysis did not provide any information about the tests you performed and mean separation methods. We can evaluate your results and interpretation without this critical information. Also, c "daily mean concentration"? | We appreciate the reviewer’s suggestion. The statistical analysis method is described in more detail in Section 2.4 and Table 1. |
| Line 145: Provide the statistical model and p-value to support this claim. | This is well noted and supplemented in Materials and Methods and Table 1 to improve clarity. |
| Line 147: Wrong. Your N and P concentration were similar for light rain and rainstorm based on your statistical analysis (which we have no idea as it is not provided). Also no difference in PP concentration among all rainfall events. | We are sorry that our original statement was not accurate. We have rephrased those inaccurate statements in the revised manuscript and respective sections of the text. |
| Line 163: You bring this "pathway analysis" from nowhere. There is nothing written about this in the materials and methods. So many terms (e.g., action coefficient) and symbols (e.g., lambda) were being forced here without any previous explanations, which could not be very clear to readers. | We thank the reviewer for this comment, and following the reviewer’s suggestion. We have now included thorough descriptions of pathway analysis to make the nitrogen and phosphorus losses process clearer. |
| Figure 5: What precipitation, event, daily or cumulative? Be specific. | Well noted, improved the terms writing to make it clearer |
| Line 191: Avoid mentioning results in the discussion. Also, you cant throw these numbers without a range and context. Numbers change depending on weather, topography and management. | noted and deleted |
| Line 194: Delete. You are repeating the same information in the next sentence. | noted and deleted |
| Line 195: You often use "significant" without a test or supporting evidence. | noted and deleted |
| Table 5: You can add the "slope/topography" of the plantations to this table, which may explain some of the observed variability. | noted and agreed |
| Line 199- it was reported | noted and corrected |
| Line 202- pollution from tea plantations in Southwest China | noted and corrected |
| Line 210- Not entirely true. Overland flow is generated when the intensity exceeds the soil's infiltration capacity. It doesn't necessarily mean that the soils are saturated at that time. Revise. | noted and corrected |
| Lines 211-213 – Merge these sentences | noted and agreed |
| Line 220- Cropping systems | noted and corrected |
| Line 236- delete the sentence "according to…." | noted and deleted |
| Reviewer #2 |  |
| 1. The key results from your abstract is not clear. You need to be very sharp on your key conclusions. | Well noted, improved the abstract writing |
| 2. Line 26-29. The conclusions and implications are not much attractive. They should be built on your key results. | Authors agree with the reviewer and have modified |
| 3. It is unclear from your introduction that why do you want to investigate Nitrogen and Phosphorus Losses? Why are they important? Why do you choose tea plant cultivation? See relevant publication, Sheteiwy et al., 2022, https://doi.org/ 10.1016/j.envpol.2022.120356; Zhou et al., https://doi.org/10.1016/j.scitotenv.2019.133845; Xu et al., https://doi.org/10.1016/j.spc.2021.04.019 | Well noted, and sentence rephrased to make it clearer |
| 4. There is quite large room to improve the writing. Not only for the writing itself but also for the writing logic. The research questions and hypotheses are not clear. | Improved writing logic, and added scientific hypotheses to the introduction |
| 5. To make your results comparable, more details on Materials and methods are required for the further evaluation. More information on the climatic, edaphic and environmental variables are required. | More details have been described in the material method |
| 6. More information on the land use history and plant and understory community composition are required. More details on the cover crop management are required. | Well noted, and a sentence has been added at the experimental design section to address this |
| 7. Relevant citations are required for your method section. | Related references have been added to the method section. |
| 8. Several unnecessary abbreviations are preventing the reading. I have to remember a lot abbreviations when reading your manuscript. | Reduce unnecessary abbreviations and enhance the readability of sentences |
| 9. You need more efforts for the results section to well focus on your key findings. This can make your results clearer. | Well noted, and sentence rephrased to make it clearer |
| 10. Some in-depth data analyses and particularly data interpretation is required to explore the underlying mechanisms. Will you compare the relative importance of these variables? Have you tried to discuss the causal relationship? | Figure 5 explains the pathway of the impact of rainfall intensity on runoff and nitrogen and phosphorus concentration in tea plantation, and the pathway coefficient reflects variable relative importance. |
| 11. You need to clearly mention the hypotheses behind your SEM. | Concerning the assumption about the nitrogen and phosphorus loss model, we clarified that we are assuming a single factor model driven by rainfall. We acknowledge that one of the specific advantages of our framework is to allow the universality of rainfall intensity in the combined effects of runoff and nitrogen and phosphorus loss concentrations. We have revised the whole section introducing the model (line 164-174). |
| 12. Tables. Decimals should be consistent across the manuscript. | noted and correction effected |
| 13. Some sentences are unnecessary long with changing focuses. It is not easy to understand these long sentences. The writing needs to be improved. | Well noted, and long sentence rephrased to make it clearer |
| 14. The main conclusions and key implications are not clear enough from your discussion. The potential mechanisms need to be discussed. What are the important biotic and abiotic factors affecting the responses? What are the key implications? How can we advance the understanding of N and P cycling from this study? For example, Luo et al., 2022, https://doi.org/10.1111/1365-2435.14178; Ren et al., 2017, https://doi.org/10.1007/s00374-017-1197-x; Jiang et al., https://doi.org/10.1016/j.fcr.2019.02.010 | We agree with the Reviewer and we now making an attempt in the Discussion (lines 205-216) to discuss some potential mechanisms of rainfall and vegetation cover on nitrogen and phosphorus loss in tea plantation. |
| 15. Fig. 1. Coordinates are required. | Fugure 1 was removed to comply with the copyright requirements of PLOS ONE. Figure 1 was described the location of the study site, which information had been described in the Materials and Methods section. We believe that removing Figure 1 will not affect the reading of the manuscript. |

Thank you for considering this paper

Yu Li (Corresponding author)

Email: (liyu083110@163.com)

Taiming Jiang (Ph.D.) (Corresponding author)

Email: (jtm532@163.com)
