# Supplementary material for: Nitrogen and phosphorus losses via surface runoff from tea plantations in the mountainous areas of Southwest China
Source: PLoS One. 2023 Jun 23;18(6):e0285112. doi: 10.1371/journal.pone.0285112 (PMC10289461; doi:10.1371/journal.pone.0285112)
Supplement: Attachment — Submitted filename: Response to Reviewers.docx [file pone.0285112.s007.docx]

***Dear reviewer,***

Thank you very much for your helpful comments on our manuscript. The changes are updated on the current manuscript. We trust that this version is in good shape for acceptance. Below are the detailed responses for each of the suggested changes.

| **Comment** | **Response** |
| --- | --- |
| **Reviewer 1** |  |
| Line 19- please delete “the results show that” | Noted and deleted |
| Line 71: when the fields were fertilized? (Follow up in discussion: Did high concentration coincide with fertilizer application dates? Some studies have shown high nutrient conc. in runoff occurred immediately following fertilization. A comment or two would be nice). | We thank the reviewer for this comment, and following the reviewer’s suggestion. We have discussed fertilizer application to concentration of nitrogen and phosphorus in surface runoff. |
| Line 79 and elsewhere – space between the word and ( | Noted and correction effected |
| Line 87 – how long the samples were stored in -4oC before being analyzed? | All runoff samples were generally analyzed within one week after collection. |
| Line 88- how did you wash the tanks. | This is well noted and supplemented in Materials and Methods to improve clarity. |
| Line 102- mention your fixed, random and repeated factors in your analysis | ANOVAs were performed with fertilizer application as the fixed factor, and rainfall intensities as the random factor, and three runoff plots and two observation years as repeated factors. |
| Line 142 – spacing was not consistent between the values and units | Noted and correction effected |
| Table 1. Describe the abbreviations in your title | Noted and correction effected |
| Line 158 – delete “clearly” | Noted and deleted |
| Line 206- did you notice that in your study? You have measured dissolve and total fractions. This could give some insights. | We thank Reviewer for critical suggestions. We discussed the differences in the dissolved fractions of nitrogen and phosphorus in runoff under different rainfall intensities. |
| Line 220- reduced tillage/conservation tillage? | Well note, it has been rephrased for clarity |
| **Editor** |  |
| Journal Requirements:  Please review your reference list to ensure that it is complete and correct. If you have cited papers that have been retracted, please include the rationale for doing so in the manuscript text, or remove these references and replace them with relevant current references. Any changes to the reference list should be mentioned in the rebuttal letter that accompanies your revised manuscript. If you need to cite a retracted article, indicate the article’s retracted status in the References list and also include a citation and full reference for the retraction notice. | Thank you for bringing this to our attention. We have thoroughly checked the reference list and confirmed that all the references cited are accurate and up-to-date. We will continue to follow the relevant standards and ensure the accuracy of the reference list. If you have any further questions or suggestions, please do not hesitate to contact us. |
| Additional Editor Comments:  The manuscript has been significantly improved, however there are still multiple areas that are not clear. Importantly, the Conclusions remain a summary of the results already presented early in the manuscript, rather than conclusions that address the implications of the study for planning or managment (eg.with respect to climate changes). the conclusions should also better address challenges and future studies. A reviewer has also listed a number or improvements that should be made. | Thank you for the feedback. We appreciate the reviewer's comments and have carefully revised the manuscript. We have worked to address the issues raised, including improving the clarity of the paper and elaborating on the implications of our findings for planning and management. As per the reviewer's suggestions, we have also made improvements to the conclusion section by better addressing the challenges and opportunities for future research. We believe that these revisions have significantly strengthened the paper and we look forward to your further feedback. |

Thank you for considering this paper

Yu Li (Corresponding author)

Email: (liyu083110@163.com)

Taiming Jiang (Ph.D.) (Corresponding author)

Email: (jtm532@163.com)
